# Factors influencing patency after percutaneous transluminal angioplasty for autogenous arteriovenous fistulae: a systematic review and meta-analysis

**DOI:** 10.1080/0886022X.2026.2647082

**Published:** 2026-04-22

**Authors:** Ya Zhan, Xinwei Fu, Guisen Li, Bin He

**Affiliations:** a Renal Department, The Third Hospital of Mianyang, Sichuan Mental Health Center, Mianyang, China; b Renal Department and Nephrology Institute, Sichuan Provincial People’s Hospital, School of Medicine, University of Electronic Science and Technology of China, Chengdu, China; c The No. 1 Department of Gerontology, The Third Hospital of Mianyang, Sichuan Mental Health Center, Mianyang, China

**Keywords:** Arteriovenous fistula, percutaneous transluminal angioplasty, patency, hemodialysis, meta-analysis

## Abstract

This systematic review and meta-analysis identified factors influencing patency after percutaneous transluminal angioplasty (PTA) for autogenous arteriovenous fistulae (AVF) in hemodialysis patients. Literature through July 2025 was searched for observational and randomized trials. Two reviewers independently performed study selection, quality assessment (Newcastle-Ottawa Scale/Cochrane tool), and data extraction. Meta-analyses used fixed-/random-effects models in R (per I^2^ statistic), with sensitivity and publication bias analyses. Fifty-two studies (*n* = 6,407) were included, 45 of high quality. For primary patency, independent risk factors were diabetes (OR = 1.02, 95% CI 1.01–1.02), lesion length >2 cm (OR = 2.87, 1.38–5.96), previous intervention (OR = 3.13, 1.69–5.79), failed AVF history (OR = 1.69, 1.22–2.35), brachiocephalic configuration (vs. radiocephalic, OR = 1.73, 1.33–2.26), vascular calcification, and multiple comorbidities. High post-PTA flow was protective, and drug-coated balloons outperformed conventional angioplasty. Secondary patency was compromised by diabetes (OR = 1.05, 95% CI 1.02–1.08), longer lesions (OR = 1.01, 95% CI 1.01–1.02), and residual stenosis (OR = 1.02, 95% CI 1.01–1.04), with procedural success protective. Intimal hyperplasia strongly predicted failure. Restenosis risk was elevated by diabetes (OR = 1.72, 95% CI 1.28–2.31) and hypertension (OR = 1.59, 95% CI 1.04–2.44), while medical therapy with nitrates (OR = 0.20, 95% CI 0.05–0.79) and higher albumin were protective. Conventional biomarkers had limited value. Long-term AVF patency after PTA depends on anatomical, historical, and pathological factors, emphasizing the need to target modifiable procedural and hemodynamic variables in clinical practice.

## Introduction

The autogenous arteriovenous fistula (AVF) represents the vascular access of choice for patients requiring long-term hemodialysis, owing to its superior long-term patency and lower complication rates compared to other modalities [1,2]. Nonetheless, AVFs are prone to complications such as stenosis, thrombosis, and dysfunction, which frequently lead to hospitalization and impose a significant socioeconomic burden on both healthcare systems and patients [3,4]. Percutaneous transluminal angioplasty (PTA) has emerged as the first-line endovascular intervention for treating these complications and salvaging dysfunctional AVF [5,6]. Despite its widespread adoption, the durability of PTA remains a major clinical concern. Post-procedural patency rates are highly variable, with reported 12-month primary patency rates hovering around only 60–70% [7,8]. This suboptimal longevity necessitates repeated interventions to maintain access patency, highlighting a critical need to identify the determinants of PTA success and failure.

Existing literature has suggested that a multitude of factors may influence post-PTA outcomes. These potential determinants encompass patient demographics (e.g., advanced age, diabetes mellitus), fistula characteristics (e.g., location, configuration), and procedural details (e.g., balloon type, use of high-pressure vs. drug-coated balloons, number of dilatations) [9–14]. However, the evidence regarding the impact of these factors is often fragmented, derived from single-center studies with limited sample sizes, and occasionally contradictory. Consequently, a definitive consensus on the key predictors of AVF patency following PTA is lacking, leaving clinicians without robust evidence to guide patient selection and procedural strategy.

Therefore, this study aims to systematically evaluate and synthesize the key factors influencing patency after AVF PTA through systematic review and meta-analysis, thereby providing evidence for optimizing clinical management and prolonging the longevity of dialysis access.

## Materials and methods

This systematic review and meta-analysis was conducted and reported in strict accordance with the Preferred Reporting Items for Systematic Reviews and Meta-Analyses (PRISMA) guidelines. The study protocol was prospectively registered on the INPLASY platform (registration number: INPLASY202590021). As our analysis was based on aggregated data from published studies, Institutional Review Board approval and informed consent were not required.

### Literature search strategy

A systematic literature search was performed across four electronic databases: PubMed, Embase, Web of Science, and the Cochrane Library, from their inception to July 2025 (Supplementary Tables S1–S4). The search was restricted to articles published in English. Searches were performed using a combination of MeSH terms and free-text keywords. The search terms included ‘percutaneous transluminal angioplasty,’ ‘drug-coated balloon,’ ‘arteriovenous fistula,’ ‘patency,’ and ‘risk factors.’ To minimize the risk of omission, we additionally manually screened the reference lists of all included studies and relevant review articles to identify any potentially eligible publications that the electronic search might have missed.

**Table 1.** Baseline characteristics of included studies.

First author, Year	Country	Study design	Sample size	Age (y)	Male (%)	Fu duration (m)	Outcome
Manninen et al, 2001 [18]	Finland	cohort	51	65 (22–80)**	29 (56.9)	16.8 (0.5–67.1)	primary patency
Clark et al, 2002 [15]	Canada	cohort	65	64 ± 15*	37 (56.9)	6–26	primary patency
Rajan et al, 2004 [16]	Canada	cohort	140	62.4 ± 15.6*	104 (74.2)	0.1-119	primary and secondary patency
Maeda et al, 2005 [17]	Japan	cohort	59	62.4 (32–86) **	44 (74.6)	0–51	primary patency
Clark et al, 2007 [19]	US	retrospective	101	58 ± 15*	66 (65.3)	0–40	primary patency
Liu et al, 2007 [20]	China	retrospective	82	61.93 ± 10.62*	32(39.0)	6	primary patency
Doi et al, 2008 [21]	Japan	cohort	54	63 ± 13*	31(57.4)	12	primary patency
Wu et al, 2009 [22]	China	cohort	100	61 ± 12*	42 (42.0)	6	restenosis
Wu et al, 2010 [23]	China	cohort	140	61 ± 13*	61(43.6)	6	restenosis
Heerwagen et al, 2012 [24]	Denmark	prospective observational	61	63 (29–89)**	40 (66.7)	0.7–31.1	primary patency
Mortamais et al, 2013 [25]	France	retrospective	75	59 ± 17*	47 (62.6)	38 ± 29	Assisted primary patency
Neuen et al, 2014 [12]	Australia	retrospective	207	57	120 (57.9)	20 (0.5-96)	primary and secondary patency
Aktas et al, 2015 [26]	Turkey	retrospective	228	56.8 ± 14.6*	129 (56.6)	28.7 (1–59)	primary and secondary patency
Romann et al, 2016 [27]	Canada	cohort	155	NR	104 (67.0)	0–42	primary and secondary patency
Wu et al, 2017 [28]	China	cohort	56	65.6 ± 12.7*	28 (50.0)	3	restenosis
Lee et al, 2018 [29]	Korea	cohort	54	65.63 (33–90)**	31(57.4)	24.62 (0.9–115.63)	primary and secondary patency
Suemitsu et al, 2018 [30]	Japan	cohort	158	71 ± 12*	96 (60.8)	6.3 (3.3–10.5)	primary patency
Wakamoto et al, 2018 [48]	Japan	RCT	71	70.0 ± 11.5*	43(60.6)	12	primary patency
Higashiura et al, 2019 [31]	Japan	cohort	61	68 (24–86)**	29 (47.5)	14 (0.4-71)	secondary patency
Manou-Stathopoulou et al, 2019 [9]	UK	cohort	124	62.6 ± 15.1*	59 (47.6)	18	primary and secondary patency
Kumbar et al, 2019 [49]	US	cohort	64	63.07 ± 11.98*	36 (56.2)	27	primary patency
So et al, 2019 [50]	Korea	cohort	73	60.4 ± 14.1*	42 (57.5)	12	Primary and secondary patency, assisted primary patency
Takahashi et al, 2020 [33]	US	cohort	210	70.65	135 (64.3)	NR	Primary and secondary patency, assisted primary patency
Miyamoto et al, 2020 [32]	Japan	cohort	159	72.0 ± 11.7*	107 (67.3)	5.4 (0.03–63.4)	primary patency
Yildiz et al, 2020 [11]	Turkey	cohort	135	58.8 ± 9.4*	101(74.8)	8.9 (4.3–13.1)	primary patency
Zhu et al, 2020 [34]	China	cohort	74	61.68 ± 11.44*	35 (47.3)	0–18	primary patency
Zhou et al, 2020 [10]	China	cohort	189	59.5 ± 12.1*	110 (58.2)	30.2 (1–58)	primary and secondary patency
Yap et al, 2021 [5]	China	cohort	307	64.3 ± 12.4*	171(55.7)	0–120	primary patency
Granata et al, 2021 [51]	Italy	cohort	162	67 ± 13*	92 (57)	0–48	secondary patency
Zheng et al, 2021 [52]	China	cohort	NR	NR	NR	6–36	primary patency
Alturkistani et al, 2022 [35]	Saudi Arabia	case-control	99	53.04 ± 11.86*	49 (49.5)	NR	restenosis
Chen et al, 2022 [36]	China	cohort	120	61.24 ± 12.12*	58 (48.3)	0–60	primary and secondary patency
Luo et al, 2022 [37]	China	cohort	128	59.1 ± 12.8*	69 (53.9)	6	restenosis
Hakki et al, 2022 [53]	UK	cohort	54	60 ± 14*	31 (58)	0–41	primary patency
Xing et al, 2023 [41]	China	cohort	199	52.9 ± 13.5*	97 (48.7)	21	primary patency
Huang et al, 2023 [38]	China	cohort	65	59.09 ± 14.94*	40 (61.5)	0–60	restenosis
Suemitsu et al, 2023 [39]	Japan	retrospective	114	72.9 ± 10.3*	79 (69.3)	8.3 (4.1–12.6)	primary patency
Zhu et al, 2023 [13]	China	cohort	149	65.4 ± 13.6*	81(54.4)	8.9 ± 3.8	primary assisted patency
Wasuthapitak et al, 2023 [40]	Thailand	cohort	62	62.2 ± 9.7*	38 (61.3)	8.1 (5.0–24.3)	primary patency
Anukanchanavera et al, 2023 [14]	Thailand	cohort	24	63.9 ± 14.2*	16 (66.67)	NR	primary patency
Chen et al, 2024 [42]	China	cohort	292	57 (49–67) **	166 (56.8)	34.8 (31.1–39.7)	primary patency
Chen et al, 2024 [43]	China	cohort	213	57.89 ± 15.06*	87 (40.8)	1–54	Primary and secondary patency, assisted primary patency
Long et al, 2024 [44]	China	cohort	137	54.37 ± 12.76*	81(59.1)	36	primary patency
Wang et al, 2024 [46]	China	cohort	185	58.51 ± 11.73*	96 (51.9)	0–25	primary patency
Spiliopoulos et al, 2024 [45]	Greece	cohort	100	67.12 ± 12.65*	84 (84)	16.4 ± 7.8	restenosis
Shahverdyan et al, 2024 [54]	US	cohort	95	64.8 ± 13.5*	69 (72.6)	POBA: 33.9^†^；DCBA:24.5^†^	primary and secondary patency
Xiong et al, 2024 [55]	China	cohort	229	57 (47–66)**	127 (55.5)	0–72	primary patency
Huo et al, 2025 [7]	China	cohort	173	63.1 ± 12.3*	90 (52.0)	0–50	primary patency
Suemitsu et al, 2025 [47]	Japan	cohort	407	72 ± 11**	261(64.1)	12.7 ± 5.6	primary patency
Xia et al, 2025 [8]	China	cohort	78	66.96 ± 12.58*	56 (71.8)	43	primary patency
Kambayashi et al, 2025 [56]	Japan	cohort	40	71 (61–80)**	35 (87.5)	0-9.9	assisted primary patency
Shintaku et al, 2025 [57]	Japan	cohort	29	72 ± 13*	18 (62.1)	0-9.9	primary patency

Y, year; m, month; Fu, follow-up; UK, United Kingdom; US, United States; POBA, plain old balloon angioplasty; DCBA, drug-coated balloon angioplasty; RCT, randomized controlled trial.

NR, Not Reported.

*Data presented as median ± standard deviation.

**Data presented as median (interquartile range).

†Follow-up reported separately for POBA and DCBA groups.

### Eligibility criteria

Inclusion Criteria: (a) Population: Adult patients (≥18 years) with ESRD undergoing PTA for the treatment of stenosis or thrombosis in an AVF. (b) Intervention: The primary intervention was PTA. (c) Outcomes: Reported factors associated with post-PTA patency (e.g., primary, assisted primary, secondary patency, assisted primary patency, or restenosis) and provided quantitative effect estimates such as Hazard Ratios (HR), Odds Ratios (OR), or Risk Ratios (RR) with corresponding 95% confidence intervals, or provided sufficient raw data for these measures to be calculated. (d) Study Design: Prospective or retrospective cohort studies, and randomized controlled trials (RCTs). (e) Publication Status: Published as a full-text article in English.

Exclusion Criteria: (a) Population: Studies focusing on arteriovenous grafts (AVGs), or studies with mixed AVF/AVG populations where AVF-specific data could not be separately extracted. (b) Study Design: Reviews, systematic reviews, meta-analyses, case series, case reports, conference abstracts, editorials, letters, animal studies, in-vitro studies, and studies for which only an abstract was available without a full text. (c) Data: Studies that did not report extractable effect estimates (OR/HR/RR) or lacked the necessary raw data for calculation. (d) Intervention: Studies where the primary intervention was not PTA (e.g., primary stenting, thrombolysis, thrombectomy, or surgical revision).

### Data extraction and quality assessment

Data extraction was performed independently and in duplicate by two reviewers (Yz and Xwf) using a pre-piloted, standardized data extraction form. Any discrepancies were resolved through consensus or, when necessary, by adjudication from a third reviewer. The extracted information included the first author, publication year, country, study design, participants’ mean age, gender distribution, sample size, factors investigated for their association with patency, and the corresponding effect estimates (e.g., HRs, ORs, RRs) with their 95% confidence intervals (95% CI). We also recorded whether these estimates were adjusted for potential confounders in multivariate analyses.

The risk of bias in included cohort studies was assessed using the Newcastle-Ottawa Scale (NOS), which evaluates studies across three domains: selection of study groups, comparability of groups, and ascertainment of the outcome of interest. The NOS awards a maximum of 9 stars, with studies scoring ≥7, 5–6, and <4 considered to be of high, moderate, and low quality, respectively. For any RCTs, the Cochrane Risk of Bias tool was used to evaluate bias across key domains, including random sequence generation, allocation concealment, blinding of participants and personnel, blinding of outcome assessment, incomplete outcome data, and selective reporting. Each domain was judged as having a ‘low’, ‘high’, or ‘some concerns’ risk of bias.

### Statistical analysis

All statistical analyses were performed using R software (version 4.3.1). A two-sided P-value of < 0.05 was defined as statistically significant for pooled estimates. For studies reporting consistent statistical measures and definitions for a given factor, we synthesized the data using meta-analysis. The choice between models was guided by the I^2^ statistic: a fixed-effects model was applied when I^2^ ≤ 50% and the *P*-value for heterogeneity was ≥ 0.1; otherwise, a random-effects model was used. The results are presented as pooled effect sizes (e.g., HR, OR) with their 95% CI. For factors that could not be quantitatively pooled due to inconsistencies in definitions, categorization, or measurement methods, a narrative synthesis was performed to systematically summarize the direction, magnitude, and statistical significance of their associations with post-procedural patency.

Publication bias was assessed quantitatively using Egger’s linear regression test and visually inspected using funnel plots when ten or more studies were available for a meta-analysis. A statistically significant threshold was set at *p* < 0.05 for Egger’s test. To evaluate the robustness of the pooled results and to identify potential sources of heterogeneity, a sensitivity analysis was conducted by sequentially excluding each individual study.

## Results

### Literature screening and characteristics of included studies

**Figure 1.**
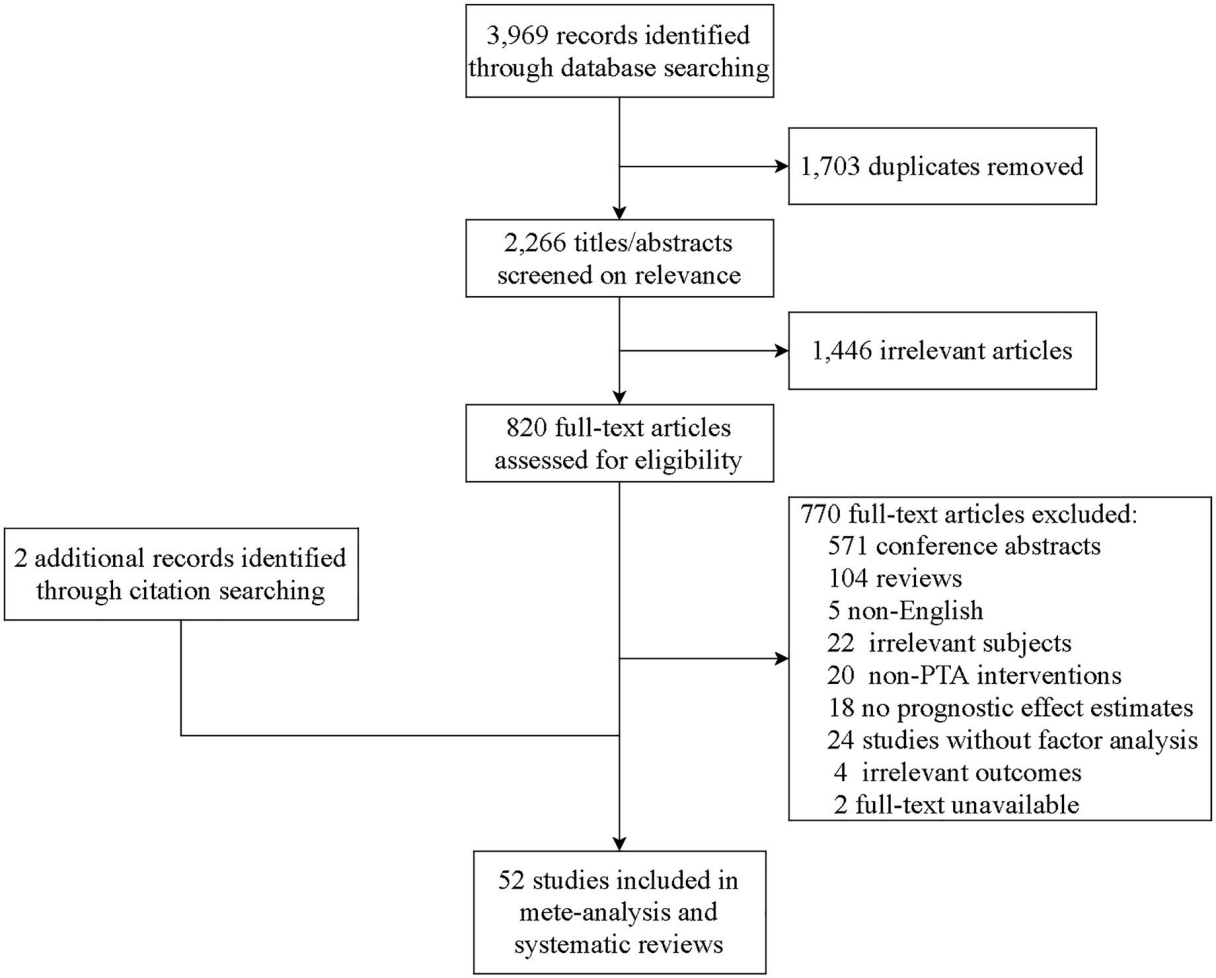
Flow diagram of study selection.

The initial database search identified 3,969 potentially relevant records. After a rigorous screening process against the eligibility criteria, 52 studies [5,7–57] were ultimately included for analysis (Figure 1). Among these, 51 studies that reported their sample size collectively involved 6,407 patients, with one study not reporting the number of participants. The included studies comprised cohort studies, case-control studies, and RCT, specifically: 50 cohort studies, one case-control study, and one RCT.

According to the NOS assessment, 45 studies were classified as high quality, while the remaining 6 were deemed moderate quality. The baseline characteristics of the included studies are summarized in Table 1. The quality assessment, detailed per study, is also available (Supplementary Tables S6–S8).

### Primary patency

#### Systematic review findings

##### Patient demographics and comorbidities

Regarding race, most studies found no significant association with primary patency, whereas one study reported a protective association for White race [9,12,19,27]. Comorbidity burden was associated with primary patency in some studies. A Charlson Comorbidity Index (CCI) ≥ 7 was defined as a definite risk factor [8], and a specific combination of diabetes, coronary artery disease (CAD), and peripheral vascular disease (PVD) was associated with a shorter time to patency loss (RR = 1.6, *p* = 0.03) [15]. Vascular calcification, including aortic arch calcification and calcification at the intervention site, was reported as a significant risk factor in two studies [5,46].

##### Hemodynamic and anatomical factors

Consistent evidence supports high post-procedural blood flow as a robust protective factor. Thresholds defining this protective effect varied slightly between studies, with flows > 500 mL/min, > 630 mL/min, and > 750 mL/min all correlating with improved outcomes [32,40]. Several anatomical characteristics were associated with loss of patency. Lesion length thresholds of > 4 cm and > 5 cm were significant predictors [12,17], as was cephalic arch stenosis [50]. Multiple stenoses, intimal hyperplasia, and luminal constrictive lesions were reported to be associated with poorer patency outcomes in several studies [30,36,39,42]. The role of central venous stenosis remains controversial, with conflicting reports in the literature [9,12,50].

##### Procedural techniques and postintervention status

Prior interventions were associated with patency outcomes. Each additional PTA increased the risk by 15%, and ≥2 prior procedures significantly elevated the hazard (HR = 1.86, *p* = 0.043) [40]. Recency of intervention was also critical, with any procedure within 90 days increasing the risk 2.52-fold [47]. Regarding device efficacy, drug-coated balloons (DCB) generally demonstrated superiority over plain old balloon angioplasty (POBA), reducing the risk by 51% [39], and providing incremental benefit over cutting balloon angioplasty (CBA) alone [14]. In one study of specific lesion types, CBA was associated with better patency than high-pressure balloons [13]. Postprocedural loss of thrill and Δ% venous intra-access pressure ratio < 33.3% were reported to be associated with poorer outcomes [19,49].

##### Biochemical markers

Most conventional biochemical markers were not significantly associated with patency outcomes in the included studies. Lipid profiles (TC, TG, HDL), serum phosphorus, and calcium-phosphorus product were not consistently associated with outcomes [5,12,20,41,43,46,52]. Findings for albumin and C‑reactive protein were inconsistent across studies [12,20,41,43,44,50,52]. One study reported that elevated pre-procedural non-classical monocyte levels were associated with increased risk [53].

##### Meta-analysis results

**Figure 2.**
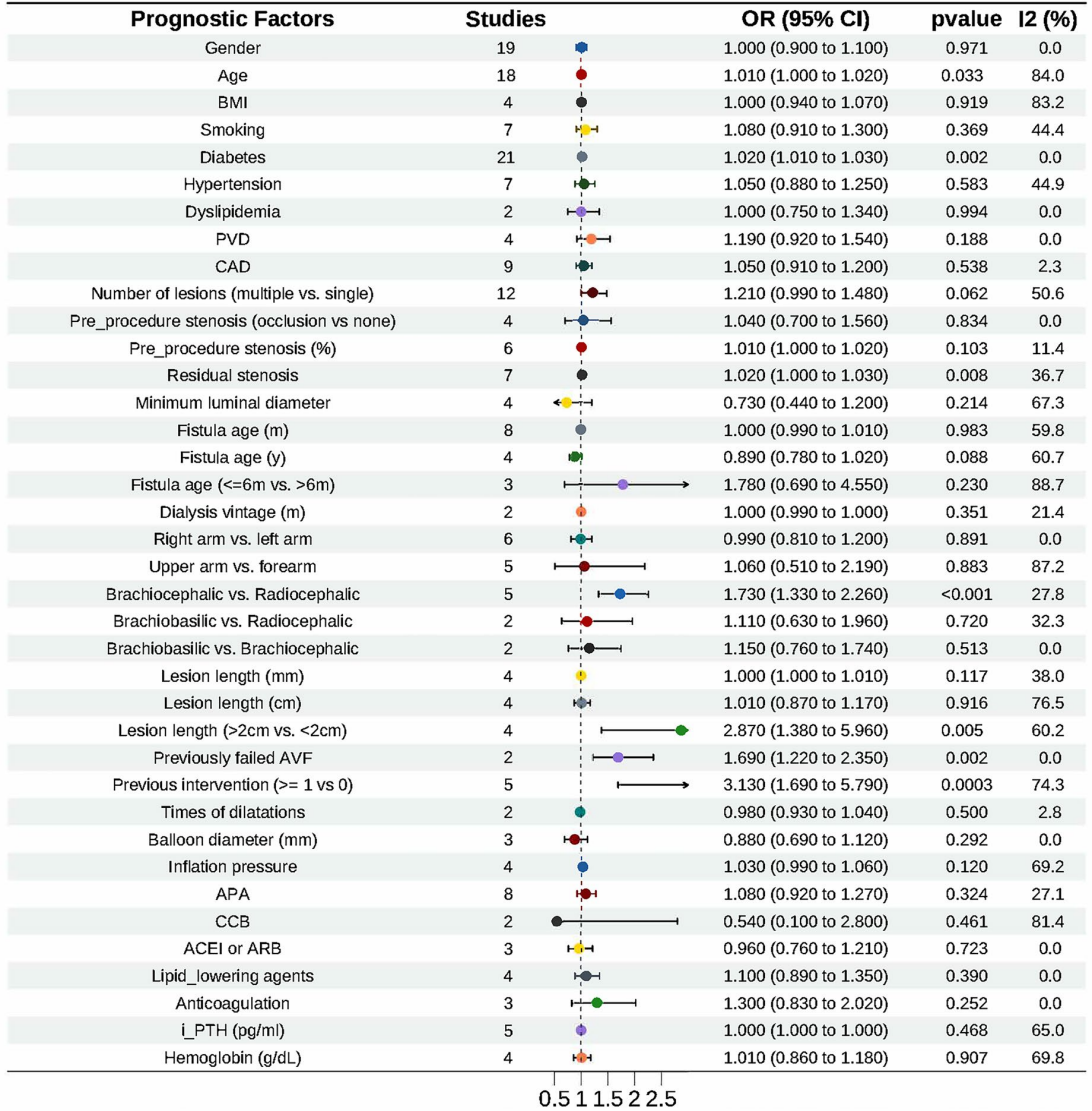
Factors influencing primary patency after PTA in AVF. The horizontal axis shows the odds ratio scale, ranging from 0.5 to 2.0. AVF, autogenous arteriovenous fistula; PTA, percutaneous transluminal angioplasty.

Diabetes mellitus (OR = 1.02, 95% CI 1.01–1.02), lesion length > 2 cm (OR = 2.87, 95% CI 1.38–5.96), previous intervention (OR = 3.13, 95% CI 1.69–5.79), and previously failed AVF (OR = 1.66, 95% CI 1.22–2.35) were associated with an increased risk of loss of primary patency. Regarding the type of arteriovenous fistula, brachiocephalic fistulae demonstrated a higher risk of impaired primary patency compared with radiocephalic fistulae (OR = 1.73, 95% CI 1.33–2.26) (Figure 2).

### Assisted primary patency

#### Systematic review findings

Cephalic arch stenosis and residual stenosis >50% were identified as significant risk factors for loss of assisted primary patency [25,50]. Findings on intimal thickness were inconsistent. One study found that greater preprocedural intimal thickness was associated with improved patency, while neither postprocedural intimal thickness nor its change demonstrated significant predictive value [13]. In contrast, another study reported that thinner intimal thickness was associated with reduced risk [56].

#### Meta-analysis results

**Figure 3.**
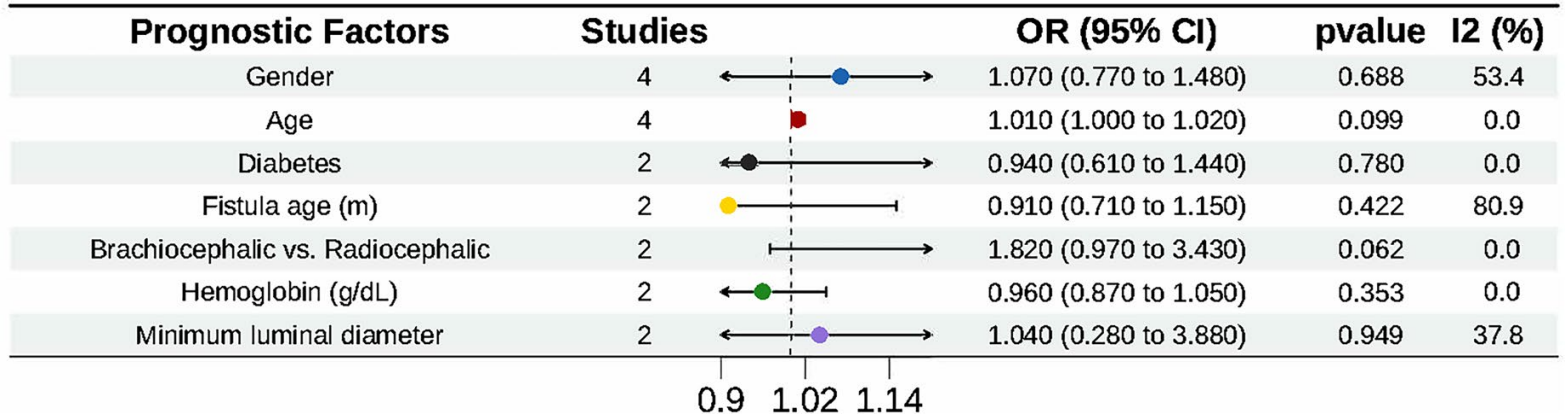
Factors influencing assisted primary patency after PTA in AVF. The horizontal axis shows the odds ratio scale, ranging from 0.5 to 2.0. AVF, autogenous arteriovenous fistula; PTA, percutaneous transluminal angioplasty.

No statistically significant associations were found between assisted primary patency and gender, age, diabetes, fistula age, hemoglobin level, or minimum lumen diameter (all *p* > 0.05). Furthermore, no significant risk difference was observed for brachiocephalic fistulae compared to radiocephalic fistulae (Figure 3).

### Secondary patency

#### Systematic review findings

One study reported a protective association for White race [9]. Investigations into serological parameters, including lipid profiles, calcium-phosphate metabolism markers, serum albumin, hemoglobin, and various inflammatory markers, uniformly demonstrated no significant association with secondary patency outcomes [12,43,50].

Both cephalic arch stenosis and long-segment lesions (>5 cm) were established as significant risk factors [12,50]. Notably, intimal hyperplasia demonstrated an exceptionally strong association with patency failure [36]. Findings on central venous lesions were inconsistent: one study reported an increased risk, whereas two studies found no significant association [9,12,50].

Among technical factors, one study reported that technical success of the initial PTA procedure was associated with better secondary patency [27].

#### Meta-analysis results

**Figure 4.**
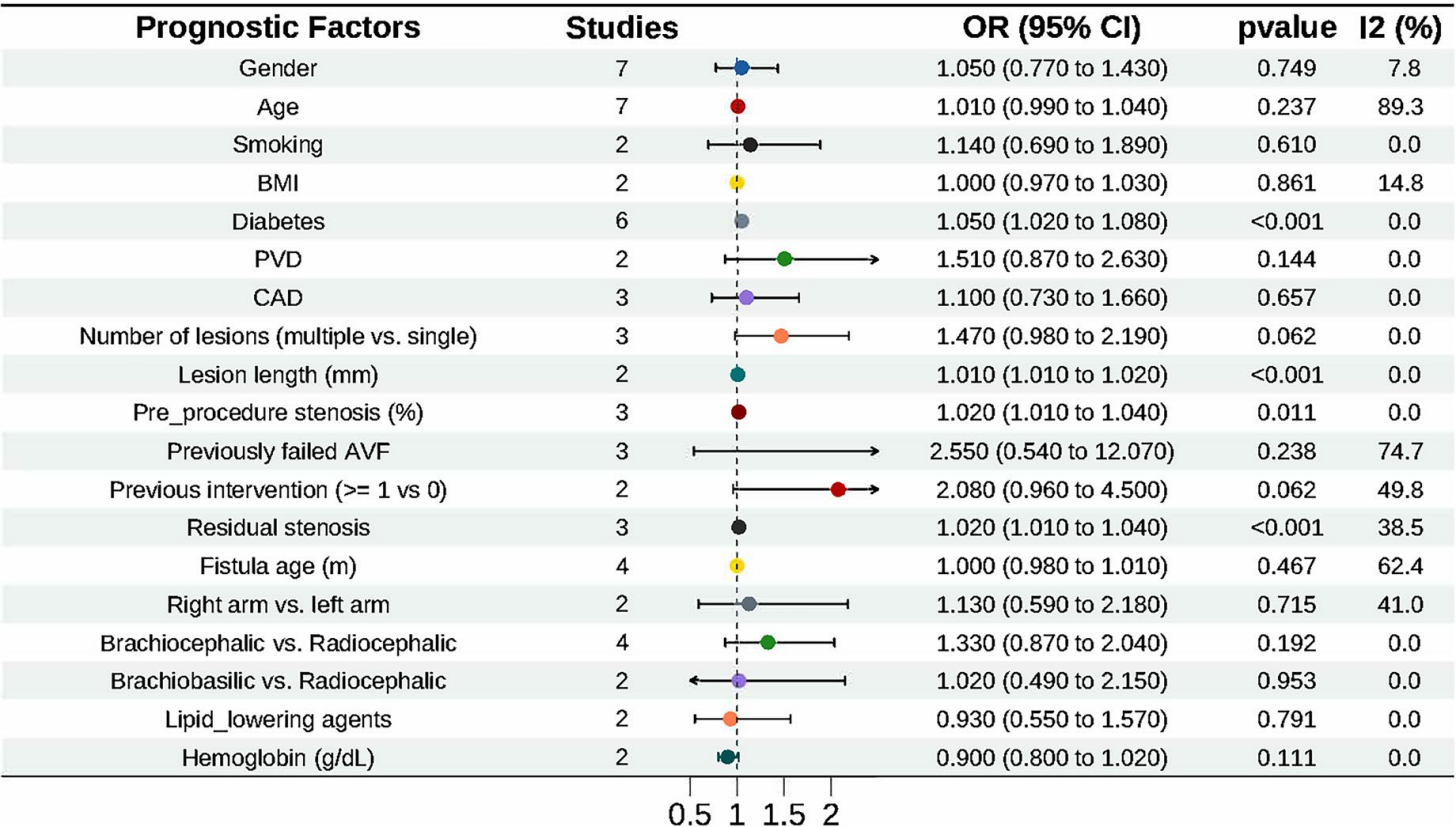
Factors influencing secondary patency after PTA in AVF. The horizontal axis shows the odds ratio scale, ranging from 0.5 to 2.0. AVF, autogenous arteriovenous fistula; PTA, percutaneous transluminal angioplasty.

Diabetes (OR = 1.05, 95% CI 1.02–1.08), lesion length per 1-mm increase (OR = 1.01, 95% CI 1.01–1.02), preprocedural stenosis severity per 1% increase (OR = 1.02, 95% CI 1.01–1.04), and residual stenosis (OR = 1.02, 95% CI 1.01–1.04) were identified as significant risk factors for loss of secondary patency (Figure 4).

### Restenosis

#### Systematic review findings

##### Vascular anatomical factors

Lesion morphology was associated with restenosis risk. A study of 128 patients found that multiple/mixed lesions, anastomotic stenosis, proximal cephalic vein stenosis, and intimal hyperplastic stenosis were all associated with substantially elevated risks [37]. Findings on lesion length were inconsistent: while one study reported a significant increase in risk per additional centimeter, two others found only non-significant trends for lengths >4 cm or >2 cm [22,28,38]. Among technical factors, one study suggested that using balloons with diameters >5 mm may reduce restenosis risk [38].

#### Biochemical markers

Among investigated biomarkers, elevated ADMA (>0.910 μM) and increased monocyte chemoattractant protein-1 (MCP-1) at two days post-PTA were identified as significant risk factors for restenosis [22,28]. One study reported an association between hypoalbuminemia (<35 g/L) and restenosis [37]. Findings for other markers were inconsistent: while higher LDL was associated with increased risk, TG and HDL showed no association [22]; findings for serum calcium and phosphorus were inconclusive [22,35,38]; and Kt/V was not predictive [22,38].

#### Meta-analysis results

**Figure 5.**
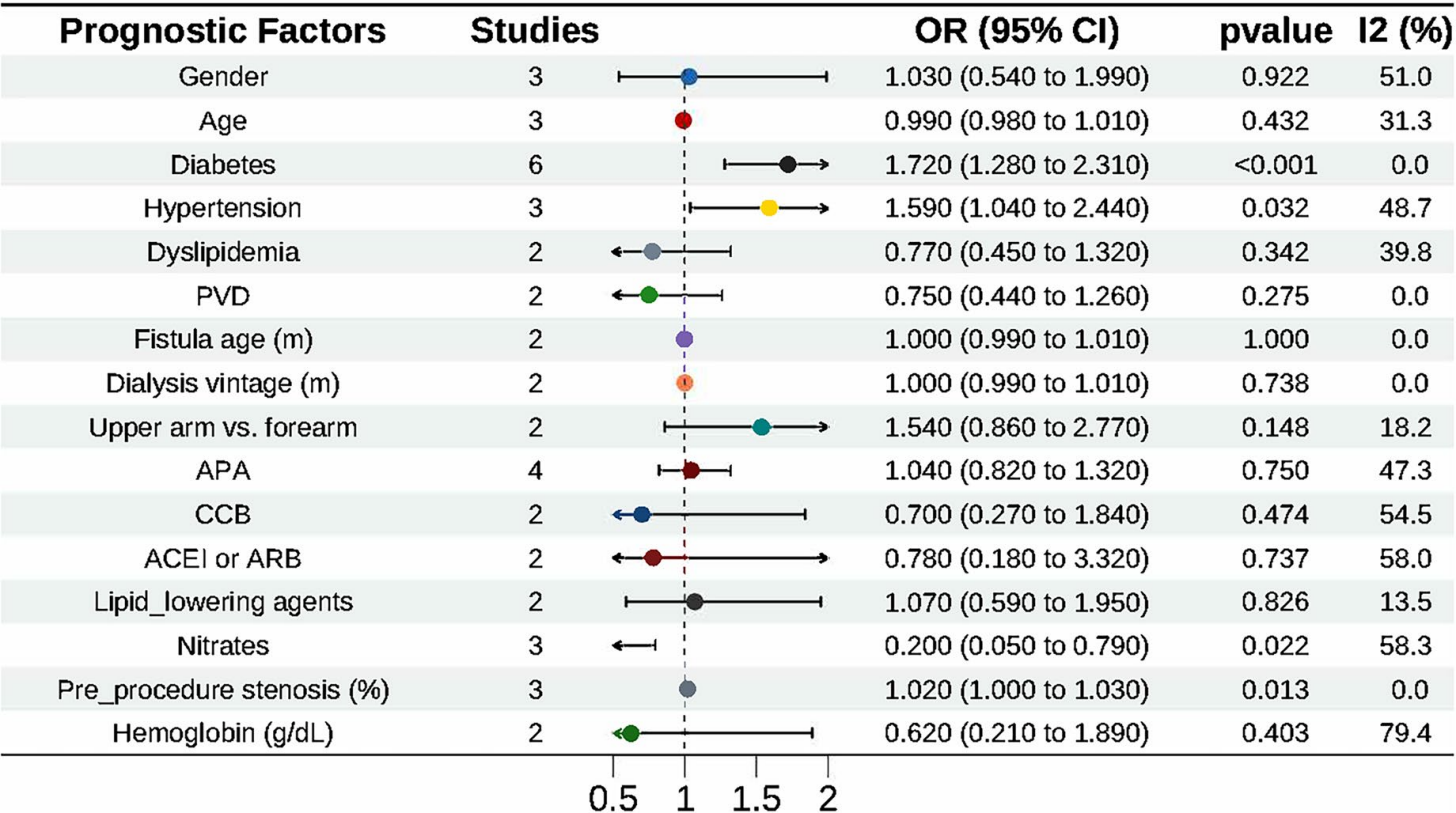
Factors influencing restenosis after PTA in AVF. The horizontal axis shows the odds ratio scale, ranging from 0.5 to 2.0. AVF, autogenous arteriovenous fistula; PTA, percutaneous transluminal angioplasty.

Diabetes (OR = 1.72, 95% CI 1.28–2.31) and hypertension (OR = 1.59, 95% CI 1.04–2.44) were associated with an increased risk of restenosis, whereas the use of nitrate medications was associated with a reduced risk (OR = 0.20, 95% CI 0.05–0.79) (Figure 5).

## Publication bias and sensitivity analysis

Sensitivity analysis indicated that the results for the majority of prognostic factors were robust. However, the pooled results for the following factors changed significantly after the exclusion of specific studies: diabetes (impact on primary and secondary patency), multiple lesions, fistula age, pre-procedure stenosis percentage, previously failed AVF, hypertension, and nitrate use (impact on restenosis) (Supplementary Table S8).

Publication bias was quantitatively assessed for factors with 10 or more studies. Egger’s test indicated potential publication bias for gender (*p* = 0.020), which was consistent with visual asymmetry in the funnel plot. No significant bias was detected for age (*p* = 0.281), diabetes (*p* = 0.561), or number of lesions (*p* = 0.657) (Supplementary Figure S1).

## Discussion

Achieving durable patency after PTA for AVF dysfunction remains a critical clinical challenge, with inconsistent post-procedural outcomes highlighting the need to clarify key influencing factors. This meta-analysis of 52 studies establishes that anatomical characteristics, procedural details, and specific patient comorbidities—rather than conventional demographics—are the primary determinants of post-PTA success, offering actionable insights to optimize clinical management.

Smoking, a well-recognized risk factor for primary AVF failure [58–60], did not emerge as a significant predictor of patency loss after PTA. This discrepancy may stem from the distinct pathological mechanisms driving postinterventional outcomes: while smoking contributes to chronic endothelial damage and atherosclerosis in native AVFs [61–63], post-PTA restenosis is predominantly mediated by local intimal hyperplasia. In this context, the systemic effects of smoking may be overshadowed by the dominant local proliferative response, suggesting a need to refocus risk stratification post-PTA on lesion-specific factors rather than smoking status alone.

The impact of diabetes on AVF patency after PTA remains a subject of debate, with several studies reporting no significant association [15–17]. Our analysis confirmed a statistically significant but modest link between diabetes and primary patency loss, with more pronounced risks for secondary patency failure and restenosis. The underlying pathophysiology likely involves diabetes‑accelerated vascular injury, endothelial dysfunction, neointimal hyperplasia, and vascular calcification [64–67]. However, sensitivity analysis indicated this finding was not robust, as statistical significance was lost upon exclusion of a key study. This highlights substantial heterogeneity across studies, likely driven by differences in diabetic severity, glycemic control, and clinical management protocols among the enrolled populations. Therefore, diabetes should not be considered a uniform risk factor. Clinical management should adopt a risk-stratified approach, prioritizing intensified surveillance and intervention for patients with poorly controlled diabetes or significant diabetic complications. A critical unanswered question is whether hyperglycemia is merely a marker of severity or a directly modifiable mediator. Consequently, prospective studies are warranted to determine if intensive glycemic control can directly improve AVF patency after PTA.

Hypertension was identified as a significant risk factor for restenosis, but the binary (yes/no) analysis of this variable represents a key limitation. The hemodynamic effects of hypertension are inherently continuous, and uncontrolled hypertension may accelerate endothelial damage and neointimal hyperplasia, while well-managed hypertension may preserve fistula function. Without granular data on blood pressure control, the true clinical significance of hypertension remains unclear, highlighting the need for future studies to quantify its impact based on treatment efficacy.

Anatomical factors are key predictors of patency after PTA. We confirmed that lesion length, location, and residual stenosis are directly associated with decreased postoperative patency, with long-segment lesions and multiple lesions having particularly significant effects. Such lesions are often accompanied by more extensive intimal hyperplasia, which not only limits balloon dilation efficacy but also increases the risk of elastic recoil [68,69]. Notably, intimal hyperplasia itself was identified as an independent risk factor in this study, providing a direct explanation for the clinical significance of these anatomical characteristics.

In recent years, suppressing intimal hyperplasia has become a research focus for improving patency. Local therapeutic approaches such as drug-coated balloons demonstrated superior efficacy compared to conventional balloons in this study, primarily through targeted inhibition of the hyperplastic process [70]. Basic research further suggests that modulating pathways such as mitochondrial fission and angiogenic factors can regulate this pathological process [71–73]. Although these advances are promising, their long-term efficacy and safety require further clinical validation. Therefore, for long-segment or multiple lesions, the use of adjunctive techniques such as cutting balloons or drug-coated balloons during intervention is recommended to achieve better luminal expansion and inhibit intimal hyperplasia.

Stenosis location is another critical variable influencing patency. Consistent evidence indicates that cephalic arch stenosis significantly increases the risk of patency loss, with its anatomical specificity (as the final outflow segment) leading to pronounced hemodynamic alterations. Although this lesion is clinically common, endovascular treatment remains technically challenging [16,74,75]. In contrast, the impact of central venous lesions remains controversial, possibly due to variations in lesion severity, sample heterogeneity, and technical differences, necessitating further research to clarify risk stratification [12,50]. Conversely, distal venous and outflow segment lesions have a weaker direct impact on patency, with most studies finding no significant association with patency decline [29,31,34]. These findings suggest that when evaluating PTA outcomes, greater attention should be paid to lesion characteristics at critical sites such as the cephalic arch.

Residual stenosis emerged as a critical modifiable risk factor, while preprocedural stenosis severity showed no significant association with outcomes. Strategies to reduce residual stenosis—such as increased dilatations, higher inflation pressure, or prolonged balloon inflation—must be balanced against the potential for increased vascular injury, which may counteract benefits [34,40,76,77]. Notably, each additional PTA procedure increased the risk of patency loss by 15%, as repeated interventions induce cumulative endothelial damage and reduce vascular remodeling capacity. This highlights the importance of optimizing initial PTA success to minimize the need for subsequent interventions.

High postoperative blood flow emerged as a robust protective factor, with flows >400 mL/min associated with improved patency and more pronounced benefits at thresholds >630 mL/min or >750 mL/min [32,40]. However, uniform ‘high-flow targets’ may not be applicable across all populations. For example, Japanese hemodialysis patients typically maintain lower average pump flow rates (approximately 200 mL/min) and may exhibit different tolerances to lower fistula flows compared to Western populations, whose dialysis blood flows are generally higher [78]. This suggests that flow targets should be individualized based on regional treatment practices and patients’ physiological characteristics. Although the definition of an ‘ideal’ flow rate remains debated, a clinical consensus is gradually emerging: extremely low postoperative blood flow (<300 mL/min) should be avoided, as retrospective studies have shown its association with a high risk of early AVF failure [79]. Therefore, from the perspective of ensuring treatment safety and accessibility, maintaining a postoperative blood flow of at least 400 mL/min may serve as a widely applicable preliminary clinical target to mitigate the risk of early dysfunction.

The role of antiplatelet and anticoagulant therapies remains uncertain. Our findings indicated no significant impact of these agents on post-PTA patency, suggesting their use should be guided by patient comorbidities (such as CAD, atrial fibrillation) rather than PTA alone. While a recent meta-analysis suggested antiplatelet therapy may benefit AVF patency, it did not specifically focus on post-PTA patients [80]. Large-scale RCTs are needed to clarify whether these medications offer net benefits in this context.

This study has some limitations. First, most included studies were observational in design, and their conclusions may be influenced by residual confounding factors that could not be fully controlled. Second, some studies did not provide multivariable-adjusted effect estimates, limiting our ability to assess potential confounding bias. Third, pooled analyses for certain risk factors exhibited substantial heterogeneity. Although subgroup and sensitivity analyses were performed to explore possible sources, this heterogeneity may reflect differences across studies in population characteristics, definitions, or measurement methods. Fourth, despite the use of funnel plot examination, for risk factors with a limited number of studies, there remains a possibility of undetected results due to publication or retrieval bias, which could affect the precision of the pooled estimates.

## Conclusions

Post-PTA AVF patency is determined by a constellation of modifiable and non-modifiable factors. High-risk patients—defined by diabetes, multiple/long lesions, residual stenosis, prior PTA interventions, or low postoperative blood flow—require risk-stratified management, including closer surveillance and early adoption of advanced therapies like DCBs. Future RCTs targeting these high-risk subgroups are essential to validate optimal treatment strategies and improve long-term dialysis access sustainability.

## Supplementary Material

Supplementary Table.docx

Supplementary Figure.docx

## Data Availability

The authors confirm that the data supporting the findings of this study are available within the article.

## Geolocation information

No.190, East Duan, Jiannan Road, Youxian District, Mianyang, Sichuan, China.
