# Supplementary material for: Factors influencing patency after percutaneous transluminal angioplasty for autogenous arteriovenous fistulae: a systematic review and meta-analysis
Source: Ren Fail. 2026 Apr 22;48(1):2647082. doi: 10.1080/0886022X.2026.2647082 (PMC13103992; doi:10.1080/0886022X.2026.2647082)
Supplement: Supplementary Table.docx [file IRNF_A_2647082_SM4652.docx]

| Table of contents | |
| --- | --- |
| Tables | Supplementary Table S1: Comprehensive search strategy for PubMed |
|  | Supplementary Table S2: Comprehensive search strategy for Cochrane |
|  | Supplementary Table S3: Comprehensive search strategy for Embase |
|  | Supplementary Table S4: Comprehensive search strategy for Web of science |
|  | Supplementary Table S5: Quality assessment of cohort studies |
|  | Supplementary Table S6. Quality assessment of case-control studies |
|  | Supplementary Table S7: Risk of bias assessment for the included randomized controlled trial |
|  | Supplementary Table S8: Sensitivity analysis of factors influencing patency of AVF after PTA |

| Table S1: Comprehensive search strategy for PubMed | | |
| --- | --- | --- |
| Search number | Query | Results |
| #1 | "Angioplasty"[Mesh] | 65,585 |
| #2 | ((((((((((((((((((((((((angioplasty[Title/Abstract]) OR (angioplasties[Title/Abstract])) OR (endoluminal repair[Title/Abstract])) OR (endoluminal repairs[Title/Abstract])) OR (percutaneous transluminal angioplasty[Title/Abstract])) OR (percutaneous transluminal angioplasties[Title/Abstract])) OR (transluminal angioplasty[Title/Abstract])) OR (transluminal angioplasties[Title/Abstract])) OR (balloon angioplasty[Title/Abstract])) OR (balloon angioplasties[Title/Abstract])) OR (PTA[Title/Abstract])) OR (drug-coated balloon[Title/Abstract])) OR (drug-coated balloons[Title/Abstract])) OR (drug coated[Title/Abstract])) OR (drug-eluting[Title/Abstract])) OR (paclitaxel-coated[Title/Abstract])) OR (paclitaxel-eluting[Title/Abstract])) OR (plain balloon angioplasty[Title/Abstract])) OR (plain balloon angioplasties[Title/Abstract])) OR (conventional balloon[Title/Abstract])) OR (conventional balloons[Title/Abstract])) OR (standard balloon[Title/Abstract])) OR (standard balloons[Title/Abstract])) OR (DCB[Title/Abstract])) OR (CB[Title/Abstract]) | 105,107 |
| #3 | #1 OR #2 | 136,370 |
| #4 | "Arteriovenous Fistula"[Mesh] | 16,289 |
| #5 | ((((((((((((((((((((((((autologous arteriovenous fistula[Title/Abstract]) OR (autologous arteriovenous fistulas[Title/Abstract])) OR (autologous arteriovenous fistulae[Title/Abstract])) OR (AVF[Title/Abstract])) OR (dialysis fistula[Title/Abstract])) OR (dialysis fistulas[Title/Abstract])) OR (dialysis fistulae[Title/Abstract])) OR (vascular fistula[Title/Abstract])) OR (vascular fistulas[Title/Abstract])) OR (vascular fistulae[Title/Abstract])) OR (arteriovenous fistula[Title/Abstract])) OR (arteriovenous fistulas[Title/Abstract])) OR (arteriovenous fistulae[Title/Abstract])) OR (hemodialysis fistula[Title/Abstract])) OR (hemodialysis fistulas[Title/Abstract])) OR (hemodialysis fistulae[Title/Abstract])) OR (autogenous AVF[Title/Abstract])) OR (arteriovenous fistulae[Title/Abstract])) OR (AV fistulas[Title/Abstract])) OR (arteriovenous access[Title/Abstract])) OR (arteriovenous accesses[Title/Abstract])) OR (hemodialysis access[Title/Abstract])) OR (hemodialysis accesses[Title/Abstract])) OR (dialysis access[Title/Abstract])) OR (dialysis accesses[Title/Abstract]) | 26,409 |
| #6 | #4 OR #5 | 34,673 |
| #7 | (((((((restenosis[Title/Abstract]) OR (re-stenosis[Title/Abstract])) OR (recurrent stenosis[Title/Abstract])) OR (post-PTA stenosis[Title/Abstract])) OR (patency[Title/Abstract])) OR (primary patency[Title/Abstract])) OR (patency failure[Title/Abstract])) OR (loss of patency[Title/Abstract]) | 70,934 |
| #8 | #3 AND #6 AND #7 | 941 |

Table S2: Comprehensive search strategy for Cochrane

| Search number | Query | Results |
| --- | --- | --- |
| #1 | MeSH descriptor: [Angioplasty] explode all trees | 5,948 |
| #2 | ((angioplasty):ti,ab,kw OR (angioplasties):ti,ab,kw OR ('endoluminal repair'):ti,ab,kw OR ('endoluminal repairs'):ti,ab,kw OR ('percutaneous transluminal angioplasty'):ti,ab,kw or ('percutaneous transluminal angioplasties'):ti,ab,kw OR ('transluminal angioplasty'):ti,ab,kw OR ('transluminal angioplasties'):ti,ab,kw OR ('balloon angioplasty'):ti,ab,kw OR ('balloon angioplasties'):ti,ab,kw or (pta):ti,ab,kw OR ('drug-coated balloon'):ti,ab,kw OR ('drug-coated balloons'):ti,ab,kw OR ('drug coated'):ti,ab,kw OR ('drug-eluting'):ti,ab,kw or ('paclitaxel-coated'):ti,ab,kw OR ('paclitaxel-eluting'):ti,ab,kw OR ('plain balloon angioplasty'):ti,ab,kw OR ('plain balloon angioplasties'):ti,ab,kw OR ('conventional balloon'):ti,ab,kw or ('conventional balloons'):ti,ab,kw OR ('standard balloon'):ti,ab,kw OR ('standard balloons'):ti,ab,kw OR (DCB):ti,ab,kw OR (CB):ti,ab,kw):ti,ab,kw) (Word variations have been searched) | 45,216 |
| #3 | #1 or #2 | 37,715 |
| #4 | MeSH descriptor: [Arteriovenous Fistula] explode all trees | 241 |
| #5 | (('autologous arteriovenous fistula'):ti,ab,kw OR ('autologous arteriovenous fistulas'):ti,ab,kw OR ('autologous arteriovenous fistulae'):ti,ab,kw OR (avf):ti,ab,kw OR ('dialysis fistula'):ti,ab,kw or ('dialysis fistulas'):ti,ab,kw OR ('dialysis fistulae'):ti,ab,kw OR ('vascular fistula'):ti,ab,kw OR ('vascular fistulas'):ti,ab,kw OR ('vascular fistulae'):ti,ab,kw or ('arteriovenous fistula'):ti,ab,kw OR ('arteriovenous fistulas'):ti,ab,kw OR ('hemodialysis fistula'):ti,ab,kw OR ('hemodialysis fistulas'):ti,ab,kw OR ('hemodialysis fistulae'):ti,ab,kw or ('autogenous avf'):ti,ab,kw OR ('arteriovenous fistulae'):ti,ab,kw OR ('av fistulas'):ti,ab,kw OR ('arteriovenous access'):ti,ab,kw OR ('arteriovenous accesses'):ti,ab,kw or ('hemodialysis access'):ti,ab,kw OR ('hemodialysis accesses'):ti,ab,kw OR ('dialysis access'):ti,ab,kw OR ('dialysis accesses'):ti,ab,kw) (Word variations have been searched) | 1,978 |
| #6 | #4 or #5 | 3,543 |
| #7 | ((restenosis):ti,ab,kw OR ('re-stenosis'):ti,ab,kw OR ('recurrent stenosis'):ti,ab,kw OR (patency):ti,ab,kw OR ('post-pta stenosis'):ti,ab,kw or ('primary patency'):ti,ab,kw OR ('patency failure'):ti,ab,kw OR ('loss of patency'):ti,ab,kw):ti,ab,kw (Word variations have been searched) | 11,312 |
| #8 | #3 and #6 and #7 | 288 |

Table S3: Comprehensive search strategy for Embase

| Search number | Query | Results |
| --- | --- | --- |
| #1 | 'angioplasty'/exp | 114,181 |
| #2 | angioplasty:ab,ti OR angioplasties:ab,ti OR 'endoluminal repair':ab,ti OR 'endoluminal repairs':ab,ti OR 'percutaneous transluminal angioplasty':ab,ti OR 'percutaneous transluminal angioplasties':ab,ti OR 'transluminal angioplasty':ab,ti OR 'transluminal angioplasties':ab,ti OR 'balloon angioplasty':ab,ti OR 'balloon angioplasties':ab,ti OR pta:ab,ti OR 'drug-coated balloon':ab,ti OR 'drug-coated balloons':ab,ti OR 'drug coated':ab,ti OR 'drug-eluting':ab,ti OR 'paclitaxel-coated':ab,ti OR 'paclitaxel-eluting':ab,ti OR 'plain balloon angioplasty':ab,ti OR 'plain balloon angioplasties':ab,ti OR 'conventional balloon':ab,ti OR 'conventional balloons':ab,ti OR 'standard balloon':ab,ti OR 'standard balloons':ab,ti OR dcb:ab,ti OR cb:ab,ti | 155,542 |
| #3 | #1 OR #2 | 201,743 |
| #4 | 'arteriovenous fistula'/exp | 45,906 |
| #5 | 'autologous arteriovenous fistula':ab,ti OR 'autologous arteriovenous fistulas':ab,ti OR 'autologous arteriovenous fistulae':ab,ti OR avf:ab,ti OR 'dialysis fistula':ab,ti OR 'dialysis fistulas':ab,ti OR 'dialysis fistulae':ab,ti OR 'vascular fistula':ab,ti OR 'vascular fistulas':ab,ti OR 'vascular fistulae':ab,ti OR 'arteriovenous fistula':ab,ti OR 'arteriovenous fistulas':ab,ti OR 'hemodialysis fistula':ab,ti OR 'hemodialysis fistulas':ab,ti OR 'hemodialysis fistulae':ab,ti OR 'autogenous avf':ab,ti OR 'arteriovenous fistulae':ab,ti OR 'av fistulas':ab,ti OR 'arteriovenous access':ab,ti OR 'arteriovenous accesses':ab,ti OR 'hemodialysis access':ab,ti OR 'hemodialysis accesses':ab,ti OR 'dialysis access':ab,ti OR 'dialysis accesses':ab,ti | 37,527 |
| #6 | #4 OR #5 | 59,721 |
| #7 | restenosis:ab,ti OR 're-stenosis':ab,ti OR 'recurrent stenosis':ab,ti OR patency:ab,ti OR 'post-pta stenosis':ab,ti OR 'primary patency':ab,ti OR 'patency failure':ab,ti OR 'loss of patency':ab,ti | 103,970 |
| #10 | #3 AND #6 AND #7 | 1,831 |

Table S4: Comprehensive search strategy for Web of science

| Search number | Query | Results |
| --- | --- | --- |
| #1 | TS=(angioplasty) OR TS=(angioplasties) OR TS=("endoluminal repair") OR TS=("endoluminal repairs") OR TS=("percutaneous transluminal angioplasty") OR TS=("percutaneous transluminal angioplasties") OR TS=("transluminal angioplasty") OR TS=("transluminal angioplasties") OR TS=("balloon angioplasty") OR TS=("balloon angioplasties") OR TS=(PTA) OR TS=("drug-coated balloon") OR TS=("drug-coated balloons") OR TS=("drug coated") OR TS=("drug-eluting") OR TS=("paclitaxel-coated") OR TS=("paclitaxel-eluting") OR TS=("plain balloon angioplasty") OR TS=("plain balloon angioplasties") OR TS=("conventional balloon") OR TS=("conventional balloons") OR TS=("standard balloon") OR TS=("standard balloons") OR TS=(DCB) OR TS=(CB) | 115,701 |
| #2 | TS=("autologous arteriovenous fistula") OR TS=( "autologous arteriovenous fistulas") OR TS=("autologous arteriovenous fistulae") OR TS=(AVF) OR TS=("dialysis fistula") OR TS=("dialysis fistulas") OR TS=("dialysis fistulae") OR TS=("vascular fistula") OR TS=("vascular fistulas") OR TS=("vascular fistulae") OR TS=("arteriovenous fistula") OR TS=("arteriovenous fistulas") OR TS=("arteriovenous fistulae") OR TS=("hemodialysis fistula") OR TS=("hemodialysis fistulas") OR TS=("hemodialysis fistulae") OR TS=("autogenous AVF") OR TS=("arteriovenous fistulae") OR TS=("AV fistulas") OR TS=("arteriovenous access") OR TS=("arteriovenous accesses") OR TS=("hemodialysis access") OR TS=("hemodialysis accesses") OR TS=("dialysis access") OR TS=("dialysis accesses") | 17,774 |
| #3 | TS=(restenosis) OR TS=( "re-stenosis") OR TS=("recurrent stenosis") OR TS=(patency) OR TS=("post-PTA stenosis") OR TS=("primary patency") OR TS=("patency failure") OR TS=("loss of patency") | 48,770 |
| #4 | #1 AND #2 AND #3 | 909 |

|  |  |  |  |  |  |  |  |  |  |  |
| --- | --- | --- | --- | --- | --- | --- | --- | --- | --- | --- |
|  |  | | | **Table S5: Quality assessment of cohort studies.** | | | |  |  |  |
|  |  |  |  |  |  |  |  |  |  |  |
|  | Selection | | | Comparability | | | Outcome | | | |
|  | Representativeness of the exposed cohort | Selection of the non exposed cohort | Ascertainment of exposure | Demonstration that outcome of interest was not present at start of study | Comparability of cohorts on the basis of the design or analysis | Assessment of outcome | Was follow-up long enough for outcomes to occur | | Adequacy of follow up of cohorts | sore |
| Manninen et al, 2001 | 1 | 0 | 1 | 1 | 2 | 1 | 1 | | 0 | 7 |
| Clark et al, 2002 | 1 | 1 | 1 | 1 | 2 | 1 | 1 | | 1 | 9 |
| Rajan et al, 2004 | 1 | 1 | 1 | 1 | 0 | 1 | 1 | | 1 | 7 |
| Maeda et al, 2005 | 1 | 1 | 1 | 1 | 2 | 1 | 1 | | 0 | 8 |
| Clark et al, 2007 | 1 | 1 | 1 | 1 | 0 | 1 | 1 | | 0 | 6 |
| Liu et al, 2007 | 1 | 1 | 1 | 1 | 0 | 1 | 1 | | 0 | 6 |
| Doi et al, 2008 | 1 | 1 | 1 | 1 | 2 | 1 | 1 | | 0 | 8 |
| Wu et al, 2009 | 1 | 1 | 1 | 1 | 2 | 1 | 1 | | 1 | 9 |
| Wu et al, 2010 | 1 | 1 | 1 | 1 | 2 | 1 | 1 | | 0 | 8 |
| Heerwagen et al, 2011 | 1 | 1 | 1 | 1 | 2 | 1 | 1 | | 1 | 9 |
| Mortamais et al, 2013 | 1 | 0 | 1 | 1 | 0 | 1 | 1 | | 1 | 6 |
| Neuen et al, 2014 | 1 | 0 | 1 | 1 | 2 | 1 | 1 | | 1 | 8 |
| Aktas et al, 2015 | 1 | 0 | 1 | 1 | 2 | 1 | 1 | | 0 | 7 |
| Romann et al, 2016 | 1 | 1 | 1 | 1 | 2 | 1 | 1 | | 0 | 8 |
| Wu et al, 2017 | 1 | 1 | 1 | 1 | 2 | 1 | 0 | | 1 | 8 |
| Lee et al, 2018 | 1 | 0 | 1 | 1 | 2 | 1 | 1 | | 1 | 8 |
| Suemitsu et al, 2018 | 1 | 1 | 1 | 1 | 2 | 1 | 1 | | 1 | 9 |
| Higashiura et al, 2019 | 1 | 0 | 1 | 1 | 2 | 1 | 1 | | 0 | 7 |
| Manou-Stathopoulou et al, 2019 | 1 | 1 | 1 | 1 | 2 | 1 | 1 | | 0 | 8 |
| Kumbar et al, 2019 | 1 | 0 | 1 | 1 | 1 | 1 | 1 | | 0 | 6 |
| So et al, 2019 | 1 | 0 | 1 | 1 | 2 | 1 | 1 | | 1 | 8 |
| Takahashi et al, 2020 | 1 | 1 | 1 | 1 | 2 | 1 | 1 | | 0 | 8 |
| Miyamoto et al, 2020 | 1 | 0 | 1 | 1 | 2 | 1 | 1 | | 0 | 7 |
| Yildiz et al, 2020 | 1 | 1 | 1 | 1 | 2 | 1 | 1 | | 1 | 9 |
| Zhu et al, 2020 | 1 | 0 | 1 | 1 | 2 | 1 | 1 | | 0 | 7 |
| Zhou et al, 2020 | 1 | 0 | 1 | 1 | 0 | 1 | 1 | | 0 | 5 |
| Yap et al, 2021 | 1 | 1 | 1 | 1 | 2 | 1 | 1 | | 0 | 8 |
| Granata et al, 2021 | 1 | 0 | 1 | 1 | 2 | 1 | 1 | | 0 | 7 |
| Zheng et al, 2021 | 1 | 0 | 1 | 1 | 2 | 1 | 1 | | 0 | 7 |
| Chen et al, 2022 | 1 | 1 | 1 | 1 | 2 | 1 | 1 | | 0 | 8 |
| Luo et al, 2022 | 1 | 1 | 1 | 1 | 2 | 1 | 1 | | 0 | 8 |
| Hakki et al, 2022 | 1 | 0 | 1 | 1 | 0 | 1 | 1 | | 0 | 5 |
| Xing et al, 2023 | 1 | 1 | 1 | 1 | 2 | 1 | 1 | | 1 | 9 |
| Huang et al, 2023 | 1 | 1 | 1 | 1 | 2 | 1 | 1 | | 1 | 9 |
| Suemitsu et al, 2023 | 1 | 1 | 1 | 1 | 1 | 1 | 1 | | 0 | 7 |
| Zhu et al, 2023 | 1 | 1 | 1 | 1 | 2 | 1 | 1 | | 0 | 8 |
| Wasuthapitak et al, 2023 | 1 | 0 | 1 | 1 | 2 | 1 | 1 | | 0 | 7 |
| Anukanchanavera et al, 2023 | 1 | 1 | 1 | 1 | 1 | 1 | 1 | | 0 | 8 |
| Chen et al, 2024 | 1 | 1 | 1 | 1 | 2 | 1 | 1 | | 1 | 9 |
| Chen et al, 2024 | 1 | 1 | 1 | 1 | 2 | 1 | 1 | | 1 | 9 |
| Long et al, 2024 | 1 | 1 | 1 | 1 | 2 | 1 | 1 | | 0 | 8 |
| Wang et al, 2024 | 1 | 1 | 1 | 1 | 2 | 1 | 1 | | 0 | 8 |
| Spiliopoulos et al, 2024 | 1 | 0 | 1 | 1 | 2 | 1 | 1 | | 1 | 8 |
| Shahverdyan et al, 2024 | 1 | 1 | 1 | 1 | 2 | 1 | 1 | | 0 | 8 |
| Xiong et al, 2024 | 1 | 0 | 1 | 1 | 2 | 1 | 1 | | 0 | 7 |
| Huo et al, 2025 | 1 | 0 | 1 | 1 | 2 | 1 | 1 | | 0 | 7 |
| Suemitsu et al, 2025 | 1 | 0 | 1 | 1 | 1 | 1 | 1 | | 0 | 7 |
| Xia et al, 2025 | 1 | 0 | 1 | 1 | 2 | 1 | 1 | | 1 | 8 |
| Kambayashi et al, 2025 | 1 | 0 | 1 | 1 | 2 | 1 | 1 | | 1 | 8 |
| Shintaku et al, 2025 | 1 | 0 | 1 | 1 | 1 | 1 | 1 | | 0 | 6 |

|  | | Table S6. Quality assessment of case-control studies | | | | | | | | | |
| --- | --- | --- | --- | --- | --- | --- | --- | --- | --- | --- | --- |
|  | Selection | | | | |  | Comparability | Exposure | | |  |
| Newcastle-Ottawa | Is the case definition adequate | | Representativeness of the cases | Selection of controls | Definition of controls |  | Comparability of cases and controls on the basis of the design or analysis | Ascertainment of exposure | Same method of ascertainment for cases and controls | Non-Response rate | sore |
| Alturkistani et al, 2022 | 1 | | 1 | 1 | 1 |  | 2 | 1 | 1 | 0 | 8 |

| Table S7: Risk of bias assessment for the included randomized controlled trial | | | | | | |
| --- | --- | --- | --- | --- | --- | --- |
| First Author, Year | Random sequence generation | Allocation concealment | Blinding of participants and personnel | Blinding of outcome assessment | Incomplete outcome data | Selective reporting |
| Wakamoto et al, 2018 | Unclear | Unclear | Unclear | Unclear | Low | Low |

Table S8: Sensitivity analysis of factors influencing patency of AVF after PTA

| Key Prognostic Factor (Outcome) | Result (Pooled OR, 95%CI) | Conclusion | Key Observation |
| --- | --- | --- | --- |
| **Primary patency** |  |  |  |
| Gender | 1.00 (0.90–1.10) | Robust | - |
| Age | 1.01 (1.00–1.02) | Robust | - |
| BMI | 1.00 (0.94–1.07) | Robust | - |
| Smoking | 1.08 (0.91–1.30) | Robust | - |
| Diabetes | 1.02 (1.01–1.03) | Non-robust | Association became non-significant after excluding Zhou et al. (2020). |
| Hypertension | 1.05 (0.88–1.25) | Robust |  |
| PVD | 1.19 (0.92–1.54) | Robust |  |
| CAD | 1.05 (0.91–1.20) | Robust |  |
| Number of lesions (multiple vs. single) | 1.21 (0.99–1.48) | Non-robust | Association strengthened (P=0.006) after omitting Yap 2021. |
| Pre−procedure stenosis (occlusion vs none) | 1.04 (0.70–1.56) | Robust | - |
| Pre−procedure stenosis (%) | 1.01 (1.00–1.02) | Robust | - |
| Residual stenosis | 1.02 (1.00–1.03) | Robust | - |
| Minimum luminal diameter | 0.73 (0.44–1.20) | Robust | - |
| Fistula age (m) | 1.00 (0.99–1.01) | Robust | - |
| Fistula age (y) | 0.89 (0.78–1.02) | Non-robust | Association strengthened (P=0.016) after omitting Suemitsu 2025. |
| Fistula age (<=6m vs. >6m) | 1.78 (0.69–4.55) | Non-robust | Association strengthened (P=0.005) after omitting Yap 2021. |
| Fistula location (right arm vs. left arm) | 0.99 (0.81–1.20) | Robust | - |
| Fistula location (upper arm vs. forearm) | 1.06 (0.51–2.19) | Robust | - |
| Fistula type (brachiocephalic vs. radiocephalic) | 1.73 (1.33–2.26) | Robust | - |
| Lesion length (mm) | 1.00 (1.00–1.01) | Robust | - |
| Lesion length (cm) | 1.01 (0.87–1.17) | Robust | - |
| Lesion length (>2cm vs. <2cm) | 2.87 (1.38–5.96) | Robust | - |
| Previous intervention (>= 1 vs 0) | 3.13 (1.69–5.79) | Robust | - |
| Balloon diameter (mm) | 0.88 (0.69–1.12) | Robust | - |
| Inflation pressure | 1.03 (0.99–1.06) | Robust | - |
| APA | 1.08 (0.92–1.27) | Robust | - |
| ACEI or ARB | 0.96 (0.76–1.21) | Robust | - |
| Lipid−lowering agents | 1.00 (0.89–1.35) | Robust | - |
| Anticoagulation | 1.30 (0.83–2.02) | Robust | - |
| i−PTH (pg/ml) | 1.00 (1.00–1.00) | Robust | - |
| Hemoglobin (g/dL) | 1.01 (0.86–1.18) | Robust | - |
| **Assisted primary patency** |  |  |  |
| Gender | 1.07 (0.77–1.48) | Robust | - |
| Age | 1.01 (1.00–1.02) | Robust | - |
| **Secondary patency** |  |  |  |
| Gender | 1.05 (0.77–1.43) | Robust | - |
| Age | 1.01 (0.99–1.04) | Robust | - |
| Diabetes | 1.05 (1.02–1.08) | Non-robust | Association became non-significant after excluding Zhou et al. (2020). |
| CAD | 1.10 (0.73–1.66) | Robust | - |
| Number of lesions (multiple vs. single) | 1.47 (0.98–2.19) | Robust | - |
| Pre−procedure stenosis (%) | 1.02 (1.01–1.04) | Non-robust | Association became non-significant after excluding Neuen et al. (2014). |
| Previously failed AVF | 0.98 (0.93–1.04) | Non-robust | Association strengthened (P=0.017) after omitting Miyamoto 2020. |
| Residual stenosis | 1.02 (1.01–1.04) | Robust | - |
| Fistula age (m) | 1.00 (0.98–1.01) | Robust | - |
| Fistula type (brachiocephalic vs. radiocephalic) | 1.33 (0.87–2.04) | Robust | - |
| **Restenosis** |  |  |  |
| Gender | 1.03 (0.54–1.99) | Robust |  |
| Age | 0.99 (0.98–1.01) | Robust |  |
| Diabetes | 1.72 (1.28–2.31) | Robust |  |
| Hypertension | 1.59 (1.04–2.44) | Non-robust | Association became non-significant after excluding Spiliopoulos et al. (2024) or Luo et al. (2022). |
| APA | 1.04 (0.82–1.32) | Robust | - |
| Nitrates | 0.20 (0.05–0.79) | Non-robust | Association became non-significant after excluding Wu et al. (2010) or Wu et al. (2017). |
| Pre−procedure stenosis (%) | 1.02 (1.00–1.03) | Robust | - |

PVD, peripheral artery disease; CAD, coronary artery disease; APA, antiplatelet agents; BMI, body mass index; AVF, autogenous arteriovenous fistulae; PTA, percutaneous transluminal angioplasty; ACEI, angiotensin-converting enzyme inhibitor; ARB, angiotensin II receptor blocker; PTH, parathyroid hormone. AVF, arteriovenous fistula; m, month; y, year.
