# Supplementary figures and images for: Factors influencing patency after percutaneous transluminal angioplasty for autogenous arteriovenous fistulae: a systematic review and meta-analysis
Source: Ren Fail. 2026 Apr 22;48(1):2647082. doi: 10.1080/0886022X.2026.2647082 (PMC13103992; doi:10.1080/0886022X.2026.2647082)

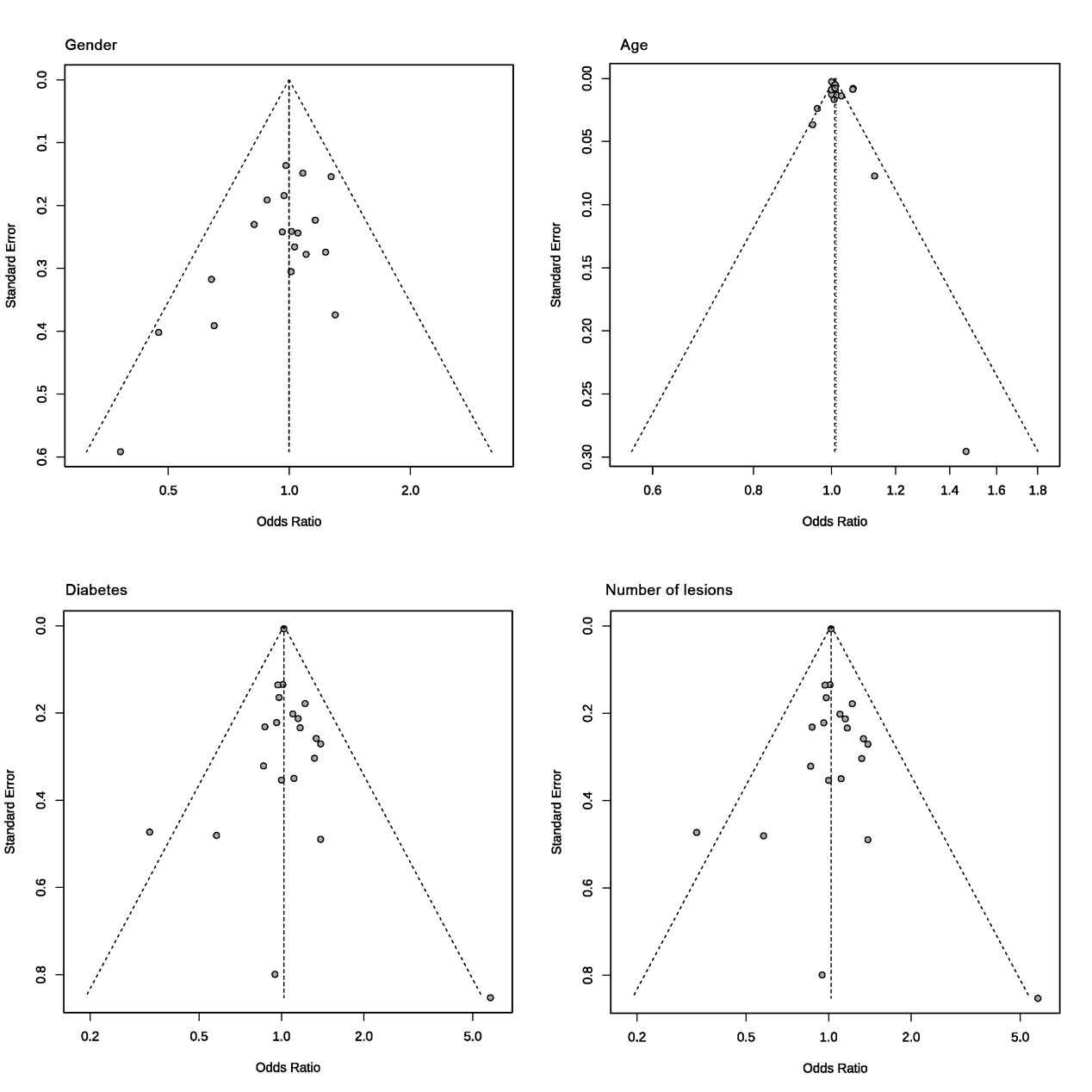


Figure S1. Funnel plots for assessment of publication bias for prognostic factors

Supplement: Supplementary Figure.docx [file IRNF_A_2647082_SM4651.docx]
